# Chemical Isotope Labeling Liquid Chromatograph-Mass Spectrometer: A Powerful Tool for Analyzing Non-Volatile Organic Acids in Baijiu

**DOI:** 10.3390/foods14061027

**Published:** 2025-03-18

**Authors:** Chi Zhao, Zhenyu Mao, Petri Penttinen, Fengju Zhang, Ling Dong, Chuan Song, Yanfei Xiong, Xiaoping Zhang, Xin Fu, Suyi Zhang, Zhihua Li

**Affiliations:** 1 Institute of Agro-Products Processing Science and Technology, Sichuan Academy of Agricultural Sciences, Chengdu 610066, China; 2 Luzhou Laojiao Co., Ltd., Luzhou 646000, China; 3 National Engineering Research Center of Solid-State Brewing, Luzhou 646000, China; 4 College of Resources, Sichuan Agricultural University, 211 Huimin Rd., Chengdu 611130, China; 5 Faculty of Agriculture and Forestry, University of Helsinki, Viikinkaari 1, 00014 Helsinki, Finland

**Keywords:** CIL-HRMS, metabolomics, NVOAs, different-flavored baijiu, biomarkers

## Abstract

Non-volatile organic acids (NVOAs) are essential to the flavor profile of Baijiu. However, the low levels and diversity of NVOAs in Baijiu make their isolation, annotation, and quantification challenging. In this study, a well-established pipeline combining chemical derivatization, isotope labeling, and high-resolution mass spectrometry with a three-tier annotation process was used to quantify NVOAs in three typical flavor types of Baijiu with high coverage and confidence. The results revealed the annotation of 56, 145, and 1277 NVOAs in Baijiu at tier 1, tier 2, and tier 3 levels, respectively. Among them, a total of 166 high-confidence NVOAs were first reported in Baijiu. Furthermore, multivariate statistical analyses indicated that abundant NVOAs could potentially be used as biomarkers to distinguish between different flavor types of Baijiu. This study provides a powerful tool for the qualification and quantification of NVOAs in Baijiu. The results will greatly expand the understanding of NVOAs in Baijiu.

## 1. Introduction

Baijiu, a Chinese distilled liquor, is a renowned beverage worldwide with a 2000-year history in Chinese culture [1]. It is an essential component of Chinese festivities and celebrations [2]. In 2022, Baijiu sales surpassed 6.7 million kiloliters valued at approximately USD 95.9 billion and were propelled by its unique manufacturing processes that produce distinct flavors [3]. The primary ingredients used in the production of Baijiu are sorghum (*Sorghum bicolor* (linn) Moench.), corn (*Zea mays* L.), wheat (*Triticum aestivum* L.), rice (*Oryza sativa* L.), and rice hulls. The key manufacturing process includes five steps: (I) production of jiuqu, (II) solid fermentation, (III) distillation, (IV) aging, and (V) blending [4]. However, the differences in raw material ratios, starters, fermentation processes, and environments have led to the diversity of Baijiu types, which are currently classified into 12 representative flavor types [5]. The essence of Baijiu’s flavor diversity can be attributed to differences in the composition and content of their aroma and taste active components [6]. Currently, Baijiu flavor component research predominantly centers on volatile components, which are immediately noticeable and have a significant impact on the aromatic qualities [2]. Research on volatile components has been extensive, with a total of 1874 compounds being identified as of 2017 [5]. In contrast, non-volatile compound research has been limited, resulting in only 144 nitrogenous, 18 polyhydroxy, and 46 non-volatile organic acids (NVOAs) being acknowledged [7]. Notably, the flavor of Baijiu is significantly influenced by non-volatile components, underscoring the need for more attention to them [2].

NVOAs have been demonstrated to contribute significantly to the flavor of Baijiu [8]. Moreover, NVOAs may serve as biomarkers for identifying the origin and flavor varieties of Baijiu [9]. However, the limited variety and low concentrations of NVOAs in Baijiu and matrix effects pose challenges for their isolation, annotation, and quantitative analysis [7]. To date, techniques employed to qualify or quantify NVOAs in Baijiu include high-performance liquid chromatography (HPLC), liquid chromatograph–mass spectrometry (LC-MS), and gas chromatography-mass spectrometry (GC-MS) [2,10,11]. HPLC has a limited coverage and high detection limit, while identification relies on standards. Furthermore, LC-MS and GC-MS (silicification derivatization) are only appropriate for identifying polar and semipolar components [11,12]. To enhance the separation and ionization of components and improve detectability in Baijiu, researchers have employed chemical derivatization techniques using LC-MS [13]. For instance, p-dimethylaminophenacyl bromide was utilized as a derivatization agent by Xie et al. to label 197 carboxylated components in Baijiu [12]. Nevertheless, annotations and quantitative analysis remain ongoing challenges.

To overcome the limitations of previous studies, this research is the first to apply a chemical isotope (^12^C/^13^C) labeling technique, developed on a high-resolution mass spectrometry platform (CIL-HRMS), in combination with a three-tiered identification process, to analyze NVOAs in three typical flavor types of Chinese Baijiu [14,15,16,17]. The findings will greatly enhance the understanding of NVOAs in Baijiu and offer novel and reliable insights into the differences in NVOAs across various Baijiu flavor types.

## 2. Materials and Methods

### 2.1. Reagents and Samples

HPLC-grade acetonitrile was obtained from Sigma-Aldrich (Shanghai, China). The DmPA-labeling kit (NMT-4167-KT, Nova Medical Testing Inc., Edmonton, AB, Canada) was used for NVOAs labeling in Baijiu.

The analysis focused on the three most popular flavor types of Baijiu in the Chinese market, including strong (Luzhoulaojiao Co., Ltd., Luzhou, China), soy sauce (Langjiu Co., Ltd., Luzhou, China), and light (Beijing Hongxin Co., Ltd., Beijing, China) flavor types. NVOAs in Baijiu were collected as described by Wang et al., (2022) [3]. Briefly, each Baijiu sample (10 mL) was initially concentrated to approximately 1 mL using a rotary evaporator and transferred to a 2 mL EP tube and then completely evaporated using high-purity nitrogen gas. The residues were subsequently resuspended in 500 μL of extraction solution (acetonitrile/water = 3:1, *v*/*v*).

### 2.2. Chemical Isotope Derivatization

Prepare independent and pooled samples according to the instructions in the labeling kit. (1) The procedure for preparing independent samples is as follows: The 25 μL of each sample was mixed with 10 μL of catalytic reagent A and 25 μL of ^12^C-labeled reagent. Subsequently, the mixture was incubated at 80 °C for 60 min, followed by the addition of 40 μL of reagent C (quenching excess labeling reagent) and incubation for 30 min to complete the chemical derivatization. (2) The procedure for preparing a pooled sample is as follows: 200 μL from each sample was taken to make a pooled sample. Subsequently, 25 μL of pooled sample was taken for ^12^C labeling following the independent sample preparation process. Meanwhile, another 25 μL of pooled sample was taken for labeling using ^13^C labeling reagent. The ^13^C-labeled and the ^12^C-labeled pooled sample were mixed in equal volumes and used as QC samples. Moreover, the ^13^C-labeled pooled samples and the ^12^C-labeled independent samples were also mixed in equal volumes for accurate relative quantitative analysis.

### 2.3. LC-MS Analysis

The NVOA separation and detection system consisted of a C18 column (150 × 2.1 mm, 1.8 μm, Agilent, Santa Clara, CA, USA), a 1290 LC system (Agilent, Santa Clara, CA, USA), and a 6546 Q-TOF mass spectrometer (Agilent, Santa Clara, CA, USA). The mobile phases A and B were water containing 0.1% formic acid and acetonitrile with 0.1% formic acid, respectively. And the flow rate and temperature of column oven were 400 μL/min and 40 °C, respectively. The gradient elution program included the following: 0–10 min, 25% B; 10–13.1 min, 25–99% B; 13.1–16 min, 99–25% B. The mass spectra were acquired at a rate of 1 Hz, with an *m*/*z* range from 220 to 1000 [15].

### 2.4. Data Processing

The raw data was converted to a .csv file using Bruker Data Analysis (version 4.4). Subsequently, IsoMS Pro (version 1.2.5, NovaMT Inc., Edmonton, AB, Canada) was used to extract peaks, align, zero-fill, filter, and impute the data. Among them, NVOAs were detected as peak pairs, i.e., light peaks from independent samples labeled with ^12^C and heavy peaks from a pooled sample labeled with ^13^C. Then, the intensity ratios between the light (^12^C) and heavy (^13^C) peaks were calculated to yield accurate relative quantitative results. The annotation of NVOAs was performed through a three-tier methodological database search: In tier 1, identifications are performed based on the MS/MS and retention time by matching with the CIL library (more than 1500 authentic metabolites); Tier 2, based on NovaMT Metabolite Database v2.0, a database of more than 9000 metabolites with predictive RT and MS/MS information, was matched for high-confidence putative identification [18]; Tier 3, identification is performed by matching with the MyCompoundID database (zero-reaction library: 8021 metabolites; one-reaction library: 375,809 metabolites; two-reaction library: 10,583,901 metabolites) based on mass [19].

### 2.5. Statistical Analysis

MetaboAnalyst 5.0 was utilized to perform principal component analysis (PCA) and partial least squares discriminant analysis (PLS-DA) [20]. The figures were generated by GraphPad Prism 9.0 software (La Jolla, CA, USA). Duncan’s multiple tests and one-way analysis of variance were conducted using the SPSS 26.0 program. The NVOAs were classified by ClassyFire software [21]. All samples were tested in three replicate experiments.

## 3. Results and Discussions

### 3.1. Profile of Non-Volatile Organic Acids in Baijiu

In this study, the NVOAs in strong, soy sauce, and light flavored Baijiu were first analyzed by CIL-HRMS with high coverage and accurate relative quantification (Supplementary Table S1). The results showed that 56 NVOAs were annotated at tier 1 based on authentic metabolites. In tier 2, according to the predicted retention time and MS/MS, a total of 145 NVOAs were annotated using the NovaMT Metabolite Database v2.0. As a result, 201 NVOAs were annotated with high confidence. For tier 3, the remaining peak pairs that were not annotated in tiers 1 and 2 were mass-matched with the MyCompoundID database, and a total of 1277 NVOAs were annotated. Compared with previous detection methods, CIL-HRMS greatly expanded the knowledge of NVOAs in Baijiu [2,7,12]. It was noteworthy that 31 and 135 NVOAs at the tier 1 and 2 levels, respectively, are reported for the first time in Baijiu [2,7,12].

**Figure 1:**
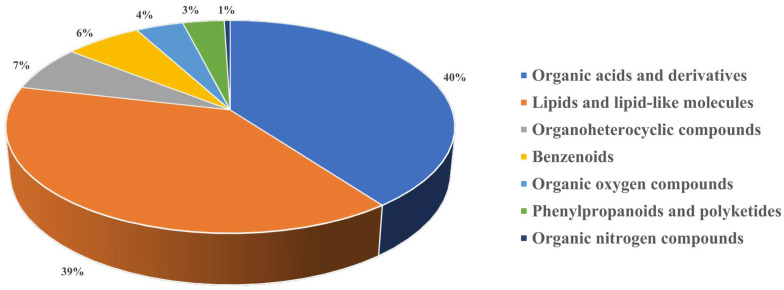
The pie graph of the number of various types of non-volatile organic acids.

Furthermore, the high-confidence annotations of NVOAs were analyzed for categorization (Figure 1). The results showed that 201 NVOAs could be categorized (superclass level) into organic acids and derivatives (*n* = 80), lipids and lipid-like molecules (*n* = 78), organoheterocyclic compounds (*n* = 14), benzenoids (*n* = 13), organic oxygen compounds (*n* = 8), phenylpropanoids and polyketides (*n* = 7), and organic nitrogen compounds (*n* = 1). Among the NVOAs in the organic acids and derivatives superclass, the subclass of amino acids, peptides, and analogues was the most abundant and has been reported to directly affect the taste and biological activity of Baijiu [22]. Specifically, L-norvaline was first detected in Baijiu, which was reported to be a non-protein branched-chain amino acid with a bitter taste [23]. Pipecolic acid, a cyclic iminic acid found in plants, is considered a neuromodulator and was also first identified in Baijiu [24]. Furthermore, at the superclass classification level, NVOAs associated with lipids and lipid-like molecules accounted for the second largest group. Notably, a significant number of fatty acyls were identified, which have not been previously reported in Baijiu [2,7,12,25]. Overall, the CIL-HRMS significantly expands the understanding of NVOAs in Baijiu.

### 3.2. Comparison of Non-Volatile Organic Acids Among Three Flavor Types of Baijiu

**Figure 2:**
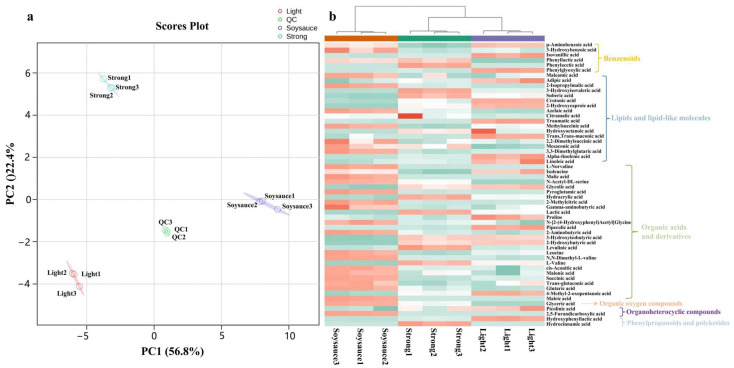
The (**a**) scores plot of principal component analysis and (**b**) clustering heatmap of different flavor types of Baijiu. QC = quality control; Light = light flavored Baijiu; Soysauce = soy sauce flavored Baijiu; Strong = strong flavored Baijiu.

Stable isotopes are considered the gold standard for mass spectrometry quantification [26]. Therefore, the accurate relative quantitative results based on isotope were used to screen for reliable biomarkers in different flavored Baijiu in the authentic metabolites annotation (tier 1). The results are presented in Figure 2a, where the QC samples are positioned at the center of the PCA score plot, with a high degree of overlap among them, indicating the excellent stability of the CIL-HRMS technique. Furthermore, PC1 and PC2 explain 56.8% and 22.4% of the total variability, respectively, with the three Baijiu flavor types clearly separated into three distinct clusters, suggesting significant differences in the NVOAs content among them. Additionally, the accurate relative quantification results, based on the peak intensity ratios of ^12^C and ^13^C, were analyzed using clustered heatmaps, which also revealed notable differences in the NVOAs across the three Baijiu flavor types (Figure 2b).

**Figure 3:**
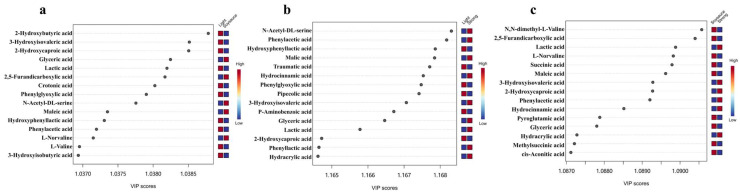
The top 15 of variable importance of the projection values of non-volatile organic acids between different flavored Baijiu. (**a**) light flavored Baijiu vs. soy sauce flavored Baijiu, (**b**) light flavored Baijiu vs. strong flavored Baijiu, as well as (**c**) strong flavored Baijiu vs. soy sauce flavored Baijiu. Light = light flavored Baijiu; Soysauce = soy sauce flavored Baijiu; Strong = strong flavored Baijiu.

Further, the differential NVOAs (DNVOAs) between different flavored Baijiu were screened using the PLS-DA model (Figure 3). The results showed that 39 NVOAs were screened as DNVOAs between the light and soy sauce flavored types of Baijiu based on the PLS-DA model (PC1 = 93% and PC2 = 1.9%; R2 = 0.99 and Q2 = 0.99; Figure 3a). Among them, 21 DNVOAs were higher in light flavored Baijiu. Notably, among the top five DNVOAs by VIP value, 2-hydroxybutyric acid, 3-hydroxyisovaleric acid, and lactic acid have been identified in Baijiu, and a previous study has shown them to be less abundant in light flavored Baijiu than in soy sauce flavored Baijiu [2]. In contrast, the contents of 2-hydroxybutyric acid, 3-hydroxyisovaleric acid, and lactic acid in light flavored Baijiu were 4.87, 5.19, and 4.87 times higher, respectively, than in soy sauce flavored Baijiu in this study. This indicates that there are still differences in NVOAs content even for the same flavor type of Baijiu, which may be due to differences in raw materials or environment. The samples of light flavored Baijiu and strong flavored Baijiu were also able to be distinguished by the PLS-DA model (PC1 = 73.2% and PC2 = 8.5%; R2 = 0.99 and Q2 = 0.97; Figure 3b). Based on the VIP values, a total of 35 NVOAs were screened as DNVOAs, among which 15 DNVOAs were higher in strong flavored Baijiu. In addition, soy sauce flavored Baijiu and strong flavored Baijiu showed significant differences in the PLS-DA scores plot (PC1 = 84.5% and PC2 = 4.7%; R2 = 0.99 and Q2 = 0.99; Figure 3c). A total of 43 DNVOAs were screened, of which 25 were found to be high in the soy sauce flavored Baijiu. Thus, the NVOAs can serve as potential biomarkers for distinguishing Baijiu of different flavor types, similar to the results of Wang et al., (2022) [3].

## 4. Conclusions

In this study, we used CIL-HRMS to quantitatively compare NVOAs in three typical flavored Baijiu with high coverage. The results showed that a total of 1478 NVOAs were annotated in Baijiu through a three-tier methodological database search. Among them, 31 and 135 NVOAs were reported for the first time in the Baijiu in the annotation results of tier 1 and tier 2 with high confidence, respectively. In addition, the results of PCA and PLS-DA showed that the abundant NVOAs have the potential to be used as biomarkers to distinguish different flavor types of Baijiu. Overall, CIL-HRMS is capable of analyzing NVOAs in Baijiu with high coverage and accurate quantification, and the results will greatly expand the understanding of NVOAs in Baijiu.

## Data Availability

The original contributions presented in the study are included in the article/Supplementary Material, further inquiries can be directed to the corresponding author.

## Supplementary Materials

The following supporting information can be downloaded at https://www.mdpi.com/article/10.3390/foods14061027/s1: Figure S1: PLS-DA scores plots of non-volatile organic acids in Baijiu with different flavor types; Table S1: List of peak pairs detected from CIL LC-MS measurement of the samples.
