# Supplementary material for: Chemical Isotope Labeling Liquid Chromatograph-Mass Spectrometer: A Powerful Tool for Analyzing Non-Volatile Organic Acids in Baijiu
Source: Foods. 2025 Mar 18;14(6):1027. doi: 10.3390/foods14061027 (PMC11941877; doi:10.3390/foods14061027)
Supplement: Supplementary file 1 [file foods-14-01027-s001.zip › foods-3334169-supplementary Figure S1.pdf]

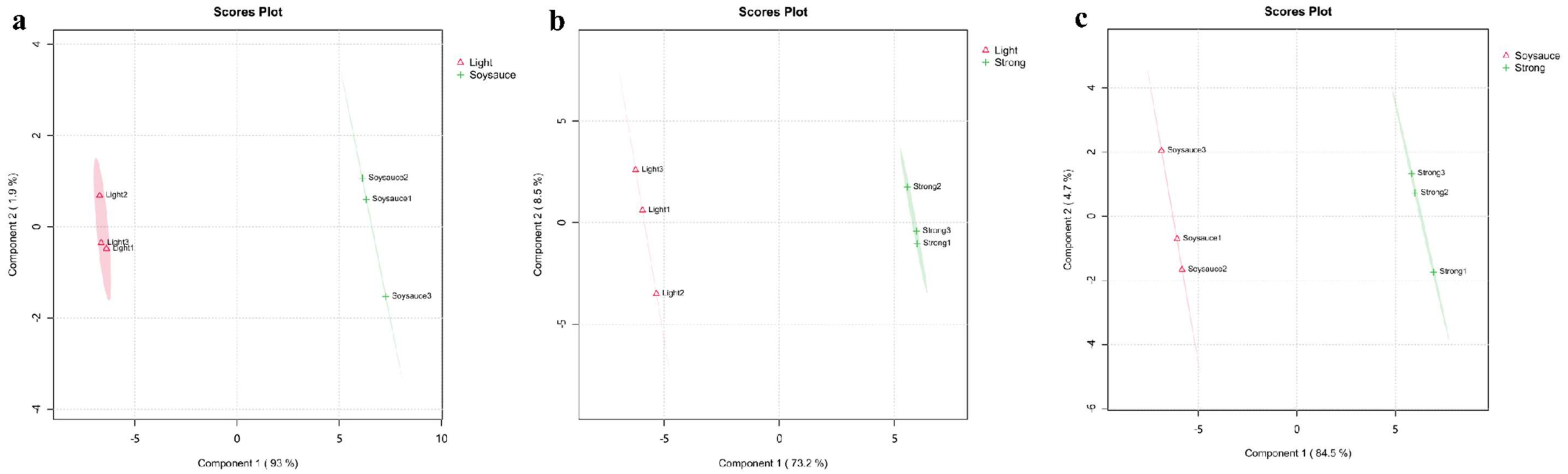

Supplementary Figure S1. PLS-DA scores plots of non-volatile organic acids in Baijiu with different flavor types. (a) light flavor Baijiu vs soysauce flavor Baijiu, (b) light flavor Baijiu vs strong flavor Baijiu, as well as (c) strong flavor Baijiu vs soysauce flavor Baijiu. Light = light flavor Baijiu; Soysauce = soysauce flavor Baijiu; Strong = strong flavor Baijiu.
